# Infertility management in resistant ovary syndrome: a review

**DOI:** 10.3389/fendo.2025.1560981

**Published:** 2025-03-18

**Authors:** Hong Yi, Lin Chen, Jinglei Zhang, Yangxing Wen, Xi Zheng, Xiaoyan Chen

**Affiliations:** ^1^ Department of Reproductive Health, Shenzhen Baoan Women’s and Children’s Hospital, Shenzhen University, Shenzhen, China; ^2^ Department of Obstetrics and Gynecology, Faculty of Medicine, The Chinese University of Hong Kong, Hong Kong, Hong Kong SAR, China; ^3^ Reproductive Medicine Center, The First Affiliated Hospital, Sun Yat-sen University, Guangzhou, China; ^4^ The First Clinical Medical College, Southern Medical University, Guangzhou, China; ^5^ Department of Obstetrics and Gynecology, Maternal-Fetal Medicine Institute, Shenzhen Baoan Women’s and Children’s Hospital, Shenzhen University, Shenzhen, China

**Keywords:** resistant ovary syndrome, follicle-stimulating hormone receptor, gene mutation, controlled ovarian stimulation, *in vitro* maturation

## Abstract

Resistant ovary syndrome is a rare endocrinological disorder characterized by elevated serum gonadotropins and normal ovarian reserves. The leading causes of this condition include *FSHR* mutations, and autoimmune disorders. Due to follicle maturation defects and anovulation, these patients have menstrual disturbances and infertility. Endocrinological disorders can be treated by regular hormone replacement therapy. However, there is no consensus regarding the infertility issues and current treatment remains experimental with controlled ovarian stimulation (COS) and *in vitro* maturation. Herein, we conducted a review of the current literature, which concludes that: 1) patients with *FSHR* mutations had very poor COS outcomes; 2) follicle-stimulating hormone and luteinizing hormone levels poorly predict COS outcomes; 3) both recombinant and urinary gonadotropins may be effective in COS; 4) the dosage of exogenous gonadotropins is not the key to successful COS; 5) *in vitro* maturation is a feasible option for patients carrying *FSHR* mutations or unsuccessful COS cycles.

## Introduction

Resistant ovary syndrome (ROS), originally described by Jones et al. in 1969 (1), is also known as ovarian insensitive syndrome and Savage syndrome. It is a rare reproductive disorder characterized by a seemingly conflicting picture of hypergonadotropic hypogonadism and normal ovarian reserves. Women with ROS generally have anovulation disorders because of ovarian hyposensitivity to endogenous gonadotropins (2), which further cause endocrinological disturbance, primary or secondary amenorrhea, and infertility. With normal ovarian reserves, ROS differs from premature ovarian insufficiency, which represents the loss of ovarian functions before the age of 40 due to the depletion of primordial follicles. However, ovarian follicle maturation is impaired due to various etiologies. Regular hormone replacement therapy can treat endocrinological and menstrual disorders, but parenthood planning remains a tricky problem. The core of fertility management is to resume follicle maturation and the current strategies include controlled ovarian stimulation (COS) (3–6), *in vitro* maturation (IVM) (7), and using donor oocytes (8). Yet, fertility management in ROS is still experimental due to its heterogenous etiology and low incidence. This review focuses on the pathogenesis and fertility management of ROS and aims to summarize the common rules of fertility management based on available evidence.

## The etiology of ROS

The etiology of ROS remains largely unclear to date. In the initial report, Jones et al. (1) proposed two hypotheses: 1) there were biologically inactive follicle-stimulating hormone (FSH) molecules in serum; and 2) the follicle apparatus itself was defective and resistant to FSH stimulation. Current studies on the pathogenesis of ROS include follicle-stimulating hormone receptor (*FSHR*) mutations and autoimmune disorders.

### *FSHR* mutations

An *FSHR*-inactivating mutation is the most studied mechanism in ROS. In 1995, Aittomaki et al. discovered the first *FSHR* mutation (c.556C>T, p.Ala189Val) in hypergonadotropic ovarian dysgenesis families (9). The mutation was located at the 7th exon of *FSHR* and caused a substitution of Ala by Val at the extracellular domain of the FSH receptor, which disrupted the cell surface targeting of the receptor and abolished cAMP production upon FSH stimulation despite the affinity with FSH remaining unaltered (9, 10). Thereafter, more than 200 mutations in *FSHR* have been discovered according to the CliniVar database, however, the clinical information was not documented in most cases. In total, 30 mutations were reported with detailed phenotypes.

These mutations involved multiple domains of the FSH receptor, and most mutations have been confirmed to impair the functions of the receptor in *in vitro* assays. In *Fshr* knock-out mouse models, preantral follicles were present in the ovaries, indicating that preantral follicle development does depend not on FSH receptors. However, no follicle progressed to the antral stages, suggesting an FSH receptor-dependent mechanism in follicle maturation (11). Similarly, equivalent small follicles were observed in ovary sections between patients with and without *FSHR* mutations, however, no follicles beyond the antral stages were observed in patients with *FSHR* mutations.

### Autoimmune disorders

In 1982, Chiauzzi et al. identified circulating immunoglobulins that inhibited FSH binding to its receptor in two patients with hypergonadotropic amenorrhea who were complicated with myasthenia gravis (12). This discovery was subsequently validated in a larger cohort consisting of 23 patients who were previously diagnosed with ROS, whereas the immunoglobulins were negative in the control group (13). Rogenhofer and colleagues reported a patient with ROS whose serum showed strong reactivity against human menopausal gonadotropins (hMG) but no reactivity to recombinant FSH (rFSH). Interestingly, the patient achieved pregnancy after controlled ovarian stimulation with a daily injection of hMG (225 IU/d) and rFSH (75IU/d) (5). Li et al. detected circulating autoimmune antibodies against FSH receptors in a patient with ROS who was successfully managed with exogenous gonadotropins and dexamethasone (4). In addition, Chitnis et al. isolated and purified an oligopeptide from human ovarian follicular fluid, which was demonstrated to inhibit the binding of FSH to ovarian granulosa cells *in vitro* and to induce atresia in developing follicles in rodent models (14). This oligopeptide may represent a potential contributing factor to gonadotropin resistance and ovarian resistance syndrome, highlighting its relevance in the pathophysiology of these conditions.

## Diagnosis of ROS

ROS shares similar clinical manifestations with premature ovarian insufficiency (POI), including menstrual disturbance (oligomenorrhea or amenorrhea), hypergonadotropinemia, decreased circulating estradiol levels, and fertility issues. However, they differ in ovarian reserves as POI represents the absolute depletion of ovarian primordial follicles at early ages whereas ROS is characterized by normal ovarian reserves, which can be distinguished by serum anti-mullerian hormone (AMH) levels and antral follicle count under transvaginal ultrasound scans. In 1972, Van Campenhout et al. (2) proposed that the diagnosis of ROS must satisfy the following three criteria: 1) endogenous hypergonadotropinemia; 2) presence of normal ovarian follicles; and 3) hyposensitivity of the ovaries to excessive stimulation of exogenous human gonadotropins. However, there were many cases that satisfied the first two criteria but showed normal ovarian responsiveness to exogenous human gonadotropins (3–5). Currently, there is no consensus regarding the diagnosis criteria of ROS but all cases to date satisfied the first two criteria.

## Infertility management strategies in ROS

Patients with ROS are primarily affected by anovulation disorder-associated menstrual disturbances, endocrinological disorders, and infertility. The first two symptoms are successfully managed with hormonal replacement therapy, whereas fertility management has been a tricky issue. Currently, there is no systematic strategy for ROS-related infertility due to heterogeneous etiology and low incidence. Potential strategies include controlled ovarian hyperstimulation (5), *in vitro* maturation (7), and using donor oocytes (8). However, these methods were attempted in separate cases with varying clinical outcomes. ROS patients who have undergone fertility management are summarized in **Table 1**, including 16 cases with *FSHR* mutations. COS and IVM were the leading strategies, and the common themes (or outcomes) of these reports are presented below.

**Table 1:** Summary of the application of controlled ovarian stimulation in resistant ovary syndrome.

Case no.	Reference	Karyotype	*FSHR* variations	Immunological disorder	bFSH IU/L	bLH IU/L	E2 pg/ml	AMH ng/ml	AFC	FSH/LH at Gn stimulation	Type of Gn	Stimulation period	Total Gn dosage	Other treatment	Oocyte yield	Clinical outcomes
1	Talbert et al., 1984 (15)	46,XX	NA	(-)	100	165	21	Normal reserves^		NA	hMG	7	NA	NA	/	No response
2	Koumantakis et al., 1997 (8)	46,XX	NA	NA	27–67	36–61	19–25	Normal reserves^		NA	hMG	15	NA	None	/	No response, pregnant using donor oocytes
3	Arici et al., 2002 (16)	46,XX	NA	Anti-ovary(-)	21–67	36–67	<25	Normal reserves^		NA	hMG	15	5,625	NA	/	No response, pregnant with donor oocytes
4		46,XX	NA	Anti-ovary(-)	74	45	21–30	Normal reserves^		NA	hMG	12	4,500	NA	/	No response
5	Mueller et al., 2003 (17)	46,XX	NA	NA	70	45	NA	Normal reserves^		NA	hMG	19	7,125	Estrogen	/	No response
										NA	hMG	17	6,375	HGF	/	No response
6	Grynberg, et al., 2013 (18)	46, XX	No mutation	None	38.4–40.3	31.7–35.7	<15	4.4–4.5	18–23	NA	rFSH	10	3,000	None	/	No response. Live birth via IVM
7	Xu et al., 2014 (19)	46, XX	NA	NA	70.3	7.6	28.6	NA	8–10	62.5/1.7	rFSH	11	2,925	None	13	Six eggs fertilized. Not pregnant after ET
										38.3/0.7	hMG	15	5,325	None	5	Clinical pregnancy
8	Rogenhofer et al., 2015 (5)	46, XX	NA	Anti-hMG (+)	58.8	23	28.7	2.1	15	NA	rFSH, hMG	14	4,295	3 HRT cycles	11	Live birth
9	Zhang et al., 2019 (20)	46, XX	NA	NA	21.6–94.9	24.7–33.8	10.7–102.8	3.2	16	NA	hMG, rFSH	15	6,000	Shift to IVM	Nine COCs	No response to COS. Live birth after shift to IVM
10	Yang et al., 2020 (21)	46, XX	NA	NA	15.6–18.4	18.1–22.8	35.7–39.5	2.5–8.7	19–20	2.9/0.8	rFSH	15	3,375	None	16	Live birth
11		46, XX	NA	NA	23.1–149.5	7.8–13.2	55.3–58.6	10.6	PCOM	13.8/2.5	hMG	9	NA	None	/	No response
										11.3/3.7	NA	16	2,400	OC	Two COCs	No response
12		46, XX	NA	NA	43.7–64.0	25.0–32.4	36.8–76.8	5.5	13–15	4.9/0.6	NA	17	6,375	None	/	No response
13	Mu, et al., 2020 (3)	46, XX	NA	NA	21.8	14.1	12	4.4	NA	NA	hMG	NA	6,075	None	/	No response
										17.2/8.9	hMG	12	5,400	OC	/	No response
										NA	hMG	12	5,400	CC	/	No response
										4.3/1.9	rFSH, hMG	18	7,275	None	/	No response
										NA	hMG	17	3,825	LE	/	Natural conception after COS
14	Samsami et al., 2020 (22)	46, XX	NA	NA	79.8–98.4	63.7–82.5	NA	3.9–6.5	NA	NA	hMG	10	4,500	NA	/	No response
15	Le et al., 2021 (23)	46, XX	None	NA	46.5	48.2	NA	2.2	14	NA	rFSH hMG	7	3,000	NA	Seven COCs	No response to COS, live birth via IVM
16	Li, et al., 2022 (4)	46, XX	No mutation	Anti-FSHR	40.8–42.4	11.5–15.1	11–15	6.2–6.3	>20	4.8/0.8	rFSH, hMG	15	3,000	None	/	No response
										7.9/0.2	rFSH, hMG	11	4,800	DXM	8	Live birth
17	Chen et al., 2022 (24)	46, XX	NA	NA	15.0	18.3	25.8	5.1	20	NA	hMG	12	2,700	None	2	No cleavage after fertilization
										13.6/12.2	hMG	12	2,700	OC	/	No response
										21.5/8.6	hMG	8	2,400	None	4	Live birth
18	Zhang et al., 2023 (25)	46, XX	NA	NA	25.8	NA	NA	1.7	7	NA	NA	8	2,400	/	4	Live birth
19		46,XX	NA	NA	25.4	NA	NA	2.5	16	NA	hMG	15	4,500	/	2	Fertilization failure
										NA	NA	11	3,300	/	Three COCs	Two embryos cryopreserved via IVM
20		46, XX	NA	NA	47.0	NA	NA	1.5	12	NA	hMG	15	5,700	/	/	No response.
										NA	NA	11	3,300	/	2	One embryo cryopreserved
										NA	hMG	8	2,400	/	0	No oocyte obtained
										NA	NA	27	12,026	/	8	Two embryos cryopreserved
21		46, XX	NA	NA	20.3	NA	NA	5.7	17	NA	NA	19	5,200	/	4	No cleavage after ICSI
										NA	hMG	7	1,800	/	/	No response
										NA	hMG	7	1,200	/	0	No oocyte retrieved
22		46, XX	NA	NA	19.1	NA	NA	2.8	9	NA	NA	21	5,700	/	8	Live birth
23		46, XX	NA	NA	31.3	NA	NA	2.3	17	NA	hMG	38	6,750	/	/	No response
24	Zhao et al., 2024 (6)	46,XX	NA	NA	18.9–28.0	36.2–41.8	56.2–143.3	5.1	19–22	18.6/10.7	hMG	14	3,675	/	2	Implantation failure
										13.3/7.3	hMG	17	4,575	/	/	No response
										4.2/2.5	hMG, rFSH	18	5,850	/	8	Live birth
25		NA	NA	NA	17.8–18.6	17.3–18.8	37.6–39.2	4.0	>24	7.8/7.3	hMG, rFSH	11	2,700	/	27	Live birth
26	Beau, et al., 1998 (26)	46, XX	c.479C>T c.1717C>T com het	NA	108	80.5	20–40	Normal reserves^		NA	rFSH	20	5,625	None	/	No response
27	Touraine, et al., 1999 (27)	46, XX	c.671A>T c.1801C>G com het	NA	63	26	10.9–21.8	NA	20–24	NA	rFSH	NA	5,625	Three HRT cycles	/	No response
28	Meduri, et al., 2003 (28)	46, XX	c.1555C>A homo	Anti-ovary -	67	21	<10	Normal reserves^		NA	rFSH	21	10,200	NA	/	No response
29	Nakamura, et al., 2008 (29)	46, XX	c.662A>T c.919G>A c.2039G>A com het	NA	11	14.2	54.4	NA	NA	NA	hMG	9	1,350	None	/	No response
										NA	hMG	6	1,800	None	/	Multi-follicular maturations, OHSS during COS
30	Desai et al., 2015 (30)	46, XX	c.1540T>C homo	NA	3.2	3.7	NA	NA	NA	NA	NA	NA	3,225	NA	29	OHSS during COS
31	Li, et al., 2016 (6)	46, XX	c.919G>A c.2039G>A com het	None	38.2–42.4	36.3–46.2	55.1–71.0	12.3	20–25	1.4/3.2	rFSH, hMG	15	4,000	Shift to IVM	Five COCs	No response to COS, live birth via IVM
32	Li et al., 2017 (31)	46, XX	c.419delA homo	NA	41.2	18.7	28.3	NA	10	NA	NA	NA	NA	NA	/	No response to high-dose FSH
33		46, XX	c.1510C>T homo	NA	83.5	46.7	84.2	NA	11	NA	NA	NA	NA	NA	/	No response to high-dose FSH
34	Flageole, et al., 2019 (32)	46, XX	c.479 T>C c.1672 A>C com het	NA	55	33	NA	3.2	19	7/6	hMG, rFSH	19	8,850	Shift to IVM	Six COCs	No response to COS, live birth via IVM
35	Khor, et al., 2020 (33)	46, XX	c.182T>A c2062C>A com het	NA	94.6	67.1	22	13.8	5–6	NA	NA	NA	NA	NA	/	No response to COS
36		46, XX	c.182T>A c.2062C>A com het	NA	85.0	65.2	17	7.1	5 ~ 6	NA	NA	NA	NA	NA	/	No response to COS
37	Kornilov, et al. 2021 (34)	46, XX	c.919A>G c.2039A>G com het	NA	25.3	29.6	15.2	38.0	45	NA	rFSH	9	450–1,125	None	/	No response
										NA	rFSH hMG	14	3,500	AI	/	No response
										NA	rFSH	17	1,700–3,400	NA	/	No response
38	Le et al., 2021 (22)	46XX, 22pstk+	c.919A>G homo	NA	91.8	35.2	NA	5.5	PCOM	NA	rFSH hMG	10	3,900	NA	3	All three oocytes were immature
										NA	rFSH hMG	7	3,000	Shift to IVM	13	No response to COS, live birth via IVM
39	Benammar et al., 2021 (35)	46, XX	c.847C>T c.1798C>A com het	NA	34.8	20	27	6.5	45	NA	NA	NA	NA	NA	/	No response to COS Live birth after shift to IVM
40	Chen et al., 2022 (36)	46, XX	c.1384G>C c.1862C>T com het	NA	36.0–37.7	21.0–21.2	41.9–45.0	2.9	6–10	NA	hMG	8	1,200	CC	/	No response
										NA	hMG	15	4,350	NA	/	No response
41	Yan et al. 2023 (37)	46, XX	c.299+2T>G homo	NA	18.4	4.8	51.6	4.7	>24	NA	hMG	NA	NA	NA	/	No response in 3 COS cycles

^normal ovarian reserves evaluated by laparoscopic biopsies; com het, compound heterozygous; homo, homozygous; HRT, hormonal replacement therapy; IVM, *in vitro* maturation; COS, controlled ovarian stimulation; OC, oral contraceptive; OC, cumulus-oocyte complex; DXM, dexamethasone; CC, clomiphene citrate.

NA, not available; hMG, human menopausal gonadotropin; rFSH, recombinant follicle-stimulating hormone; COC, cumulus-oocyte complex; OC, oral contraceptive; DXM, Dextromethorphan; COS, controlled ovarian stimulation; IVM, in vitro maturation; ICSI, intracytoplasmic sperm injection; HRT, hormonal replacement therapy; OHSS, ovarian hyperstimulation syndrome.

### Patients with confirmed *FSHR* mutations had very poor COS outcomes

To date, more than 30 inactivating mutations of *FSHR* with demonstrated phenotypes in women have been discovered (38, 39), most of which have been confirmed to undermine FSH receptor functions by *in vitro* assays, and there were 16 cases (cases 26–41) who received COS (**Table 1**) for infertility management. Excessive exogenous gonadotropins were administrated but dominant follicles were observed in only two patients (cases 30 and 38) (23, 30). Case 30 had a homozygous c.1540 T>C mutation of the *FSHR* and she developed ovarian hyperstimulation syndrome during ovarian stimulation. However, *in vitro* assays confirmed that the mutation enhanced the function of FSH receptors, indicating that it is an activating mutation. In addition, she sought assisted reproductive technology (ART) treatment due to tubal and male factors rather than ovulatory disorders (30). Similar findings were also observed in case 29, a patient with compound heterozygous c.662A>T, c.919G>A, and c.2039G>A mutations in *FSHR* who also developed ovarian hyperstimulation syndrome during ovarian stimulation (29), but no experimental evidence regarding the impact of these mutations on receptor functions was available. Interestingly, in case 38, a patient with a homozygous c.919A>G mutation in *FSHR* was unresponsive to exogenous FSH in a 7-day stimulation according to follicle size under transvaginal ultrasound scans and serum estradiol levels, but four mature eggs, along with four metaphase I (MI) oocytes and five germinal vesicles (GV), were retrieved after being triggered with hCG for IVM purposes (23). This result may be explained by residual FSHR function despite its mutations; however, no experimental evidence was available.

Furthermore, there were many patients with *FHSR* mutations for whom ovarian stimulation was not performed because of their younger age or lack of pregnancy desire despite clinical and experimental evidence showing that mutations compromise FSHR functions (30, 33, 40–46). Among the cases where ovarian stimulation with exogeneous gonadotropins was effective, genetic tests regarding *FSHR* were negative (4, 18) or were not performed (5, 6, 25). It is worth noting the obviously divided ovarian responsiveness to endogenous and exogenous gonadotropins in some cases (cases 7, 8, 10, 16, 24, and 25; **Table 1**). These cases were normal or high ovarian responders when exogenous gonadotropin was administrated despite high endogenous gonadotropin levels (4–6, 19, 21). Therefore, abnormalities of the endogenous gonadotropins may be the cause, as proposed by Jones and Moraes in the initial report (1). In conclusion, for patients with homozygous and compound heterozygous *FSHR* inactivating mutations, a trial of controlled ovarian stimulation is of limited value.

### The prognostic value of FSH and luteinizing hormone levels for COS outcomes

For those without an *FHSR* mutation or where genetic tests are not available, ovarian stimulation is still worth trying for pregnancy management. Many women have achieved pregnancies and live births with the use of appropriate ovarian stimulation. According to Huang et al., basal FSH levels were negatively associated with COS outcomes. The higher the FSH levels, the poorer the outcomes (47). However, the conclusion was limited by its sample size, which included only six patients, five of whom were carrying *FSHR* mutations.

A downregulation protocol was the most popular protocol in patients with ROS, intended to improve ovarian responsiveness to FSH stimulation by inhibiting serum gonadotropin levels (48), and similar strategies included pretreatment with oral contraceptives (OC). However, it seems that FSH and luteinizing hormone (LH) levels (after pretreatment) were not correlated with COS outcomes in previous studies. In case 7 (**Table 1**), for example, FSH/LH levels before and after downregulation were as high as 70.3/7.6 IU/L, 62.5/1.7 IU/L, and 38.3/0.7 IU/L, respectively. However, 13 and 5 oocytes were obtained after ovarian stimulation with exogenous gonadotropins (19). Similar results were also observed in other cases (cases 17 and 24; **Table 1**) (6, 24). However, successful inhibition of serum gonadotropins does not necessarily lead to improved ovarian sensitivity to FSH stimulation and favorable oocyte yields, such as in cases 12, 13, and 16 (**Table 1**) (3, 4, 21). There were also successful COS cycles after successful FSH/LH inhibition and failed COS cycles after failed FSH/LH inhibition (**Table 1**). Unfortunately, the serum gonadotropin levels at the time of exogenous FSH stimulation were not documented in most cases, leading to a lack of data for further analysis. Given the complexities of the pathogenesis of ROS and insufficient etiology investigations (e.g. *FSHR* mutations), downregulation may be attempted when considering COS, whereas the failure to inhibit serum FSH and LH levels can then lead to ovarian stimulation.

### Recombinant and urinary gonadotropins can be equally effective in COS

Among the cases successfully managed by COS, hMG (cases 7 and 17; **Table 1**), rFSH (cases 6 and 10; **Table 1**) and the combination of both (cases 8, 16, 24, and 25; **Table 1**) have been attempted, leading to a median (P25, P75) yield of 4.0 (2.0, 12.0) oocytes. In addition, hMG and rFSH were used in two individual cycles in case 7, and mature oocytes were obtained in both cycles (19). Interestingly, Rogenhofer et al. detected serum antibodies against hMG but not rFSH in a patient with ROS. Nevertheless, 11 oocytes were retrieved after stimulation with hMG (225 IU/d) and recombinant FSH (75 IU/d) (5). Given the complex mixture of hMG (49), the antibodies concerned may target other ingredients rather than FSH. Meanwhile, both recombinant hCG and urinary hCG had been proven to be effective if dominant follicles were observed after FSH stimulation. Therefore, both recombinant and urinary gonadotropins are effective in patients with resistant ovary syndrome.

### The dosage of exogenous gonadotropins is not the key to successful COS

Excessive gonadotropins were prescribed in several cases (cases 3–5, 9, 12–14, 20, 23, 26-28, 31, 34, and 40; **Table 1**) (7, 16, 17, 21, 22, 25–28, 32, 36), but in vain. There were 19 successful COS cycles (defined by the retrieval of mature oocytes) and 33 failed COS cycles (**Table 1**), respectively. The median (P25, P75) stimulating duration was 13.00 (10.25, 15.50) vs. 15.00 (10.00, 17.00) (*p* = 0.529, by Mann Whitney test) days, respectively. Furthermore, the total gonadotropin (FSH) dosage per cycle was 3,375 (2,700, 4,800) vs. 4,500(3,000, 6,038) (*p* = 0.190, by Mann Whitney test) IU, respectively. The total dosages of FSH prescribed in the failed cycles were surprisingly higher than the successful cycles, though not statistically significant. In addition, several cycles were canceled due to a lack of follicle growth after a short period of ovarian stimulation, which led to the underestimation of the actual dosage of gonadotropins in this group. Therefore, simply increasing the dosage of gonadotropins does not necessarily improve COS outcomes, while investigations into etiologies may provide more clues for further management (e.g., genetic evaluations). For example, similar dosages of exogenous FSH [3,500 (1 700, 5 625)] were prescribed to those patients with *FSHR* mutations^1^ when compared with those successful cycles, but seldomly did it work. Given the complexities of ROS, simply increasing the dosage of gonadotropins in COS is not recommended.

### IVM is a feasible option for ROS

*In vitro* maturation, as a method that supports immature GV-stage cumulus-oocyte complexes (COCs) from antral follicles to grow into the metaphase II (MII) stage, may serve as a final resort for fertility management in ROS (50). Several live births have been reported in patients with this condition who showed no response to conventional ovarian stimulation (7, 18, 21, 25, 32, 51). Currently, 12 patients have achieved live births via IVM, including 10 who were resistant to exogenous gonadotropin stimulation (**Table 2**). On average, 10 COCs were retrieved per IVM cycle, yielding an overall maturation rate of 41.51% per cycle and a live birth rate of 54.54% per patient (**Table 2**). In addition, there were serval patients who still had their embryos cryopreserved, and it is likely that the number of live births will increase (25). Eftekhar et al. also reported a series of cases managed with IVM, however, these patients seemed unlikely to have resistant ovary syndrome as their FSH levels were within the normal range (52). Overall, IVM is a feasible option for patients with *FSHR* mutations or unsuccessful COS cycles.

**Table 2:** *In vitro* maturation for patients with resistant ovary syndrome.

Reference	No.	COS outcomes	COCs	MII via IVM	Fertilization	Usable Embryo	ET	Clinical Outcomes
Grynberg et al., 2013 (18)	1	No response	15	12	7	3 D2	3	Singleton live birth
Li et al., 2016 (7)	2	No response	5	3	3	3D3	2D3	Singleton live birth
Flageole et al., 2019 (32)	3	No response	6	4	4	1 D3	1	Singleton live birth
Zhang et al., 2019 (20)	4	No response	9	9	NA	3 blastocysts	1	Singleton live birth
Yang et al., 2020 (21)	5	NA	10	NA	4	4	2	Singleton live birth
	6	No response	5	NA	0	/	/	Fertilization failure
			9	1	1	0	/	No usable embryo
Benammar et al., 2021 (35)	7	No response	16	7	7	2 D5	1	Singleton live birth
							1	Not pregnant
Le et al., 2021 (23)	8	No response	7	3	2	2 D2	2	Singleton live birth
	9	No response	9	2	3* + 2#	3D3* + 2D2#	2D3	Singleton live birth
Kornilov et al., 2021 (34)	10	No response	10	6	4	3D5	1	Singleton live birth
Zhang et al., 2023 (25)	11	No response	3	NA	2	2	0	Two embryos cryopreserved
	12	Limited response	2	0	0	/	/	No mature oocytes after IVM
	13	No	1	0	/	/	/	No mature oocytes after IVM
Galvão et al., 2018 (51)	14	No response	3	0	/	/	/	No mature oocytes after IVM
			3	0	/	/	/	No mature oocytes after IVM
	15	Yes^	4	2		1D3		One embryo cryopreserved
	16	NA	0	/	/	/	/	No COCs obtained
			3	0	/			No mature oocytes after IVM
	17	No response	5	5	/	1 D3	1	Not pregnant
	18	No response	19	2	/	0		No usable embryo
			28	1	/	1 D3	/	First-trimester miscarriage
	19	NA	21	11	/	8 D3	8	Not pregnant in six FET cycles
	20	NA	3	0	/	/	/	No mature oocytes after IVM
			33	4	/	1 D3	1	Singleton live birth
	21	No response	12	10	/	2 D3	2	Not pregnant
			8	6	6	0	/	Poor-quality embryos
			2	2	/	1 D3	1	Not pregnant
			5	4	2	1 D3	0	Embryo degradation
			5	5	1	0	/	No usable embryos
			11	5	/	2 D3	0	Embryo degradation
			7	3	/	2 D3	2	Singleton live birth
	22	No response	6	0	/	/	/	No mature oocytes after IVM
			14	3	3	3 D3	1	Live birth
							1	Biochemical pregnancy
							1	Not pregnant
			30	3	NA	2 D3	1	Biochemical pregnancy
							1	Not pregnant
			35	9	NA	3 D3	1	First-trimester miscarriage, two poor-quality embryos degraded
			14	4	0	/	/	No fertilization
			5	3	NA	2 D3	1	Twins live birth

*in vivo maturation; ^#^ *in vitro* maturation; ^responsive in one out of three COS cycles.

## Conclusions

Patients with *FSHR* mutations had very poor COS outcomes, making it not worthy of a trial. For those without an *FSHR* mutation, COS is worth trying, with either recombinant or urinary gonadotropins at approximate dosages, whereas FSH and LH levels poorly predict COS outcomes. Furthermore, IVM is a feasible option for patients carrying *FSHR* mutations or with unsuccessful COS cycles.

## Footnotes

1 Case 30 was excluded from calculation due to the activating nature of the mutation.
